# Survival After Lobectomy vs. Sublobar Resection for Stage IA Large-Cell Neuroendocrine Carcinoma of the Lung: A Population-Based Study

**DOI:** 10.3389/fsurg.2022.856048

**Published:** 2022-03-15

**Authors:** Xiangyang Yu, Mengqi Zhang, Feifei Wang, Xiaotong Guo, Kai Ma, Lixu Wang, Hongbo Zhao, Hua Xiao, Chujian Huang, Longde Du, Ran Jia, Yikun Yang, Lanjun Zhang, Zhentao Yu

**Affiliations:** ^1^Department of Thoracic Surgery, National Cancer Center/National Clinical Research Center for Cancer, Cancer Hospital & Shenzhen Hospital, Chinese Academy of Medical Sciences and Peking Union Medical College, Shenzhen, China; ^2^Department of Pathology, Shenzhen Maternity and Child Healthcare Hospital, Shenzhen, China; ^3^Department of Radiology, National Cancer Center/National Clinical Research Center for Cancer, Cancer Hospital & Shenzhen Hospital, Chinese Academy of Medical Sciences and Peking Union Medical College, Shenzhen, China; ^4^State Key Laboratory of Oncology in South China, Department of Thoracic Surgery, Collaborative Innovation Center for Cancer Medicine, Sun Yat-sen University Cancer Center, Guangzhou, China

**Keywords:** pulmonary larger cell neuroendocrine carcinoma, SEER, sublobar resection, lobectomy, survival

## Abstract

**Objective:**

Due to the low incidence of pulmonary large cell neuroendocrine carcinoma (LCNEC), the survival analysis for comparing lobectomy and sublobar resection (SLR) for stage IA LCNEC remains scarce.

**Methods:**

Patients diagnosed with pathological stage IA LCNEC between 1998 and 2016 were extracted from the Surveillance, Epidemiology, and End Results (SEER) database. The oncological outcomes were cancer-specific survival (CSS) and overall survival (OS). Kaplan–Meier analysis and Cox multivariate analysis were used to identify the independent prognostic factors for OS and CSS. Furthermore, propensity score matching (PSM) was performed between SLR and lobectomy to adjust the confounding factors.

**Results:**

A total of 308 patients with stage IA LCNEC met the inclusion criteria: 229 patients (74.4%) received lobectomy and 79 patients (25.6%) received SLR. Patients who underwent SLR were older (*P* < 0.001), had smaller tumor size (*P* = 0.010), and less lymph nodes dissection (*P* < 0.001). The 5-year CSS and OS rates were 56.5 and 42.9% for SLR, and 67.8 and 55.7% for lobectomy, respectively (*P* = 0.037 and 0.019, respectively). However, multivariate analysis did not identify any differences between the SLR group and lobectomy group in CSS (*P* = 0.135) and OS (*P* = 0.285); and the PSM also supported these results. In addition, the age at diagnosis and laterality of tumor were identified as significant predictors for CSS and OS, whereas the number of lymph nodes dissection was a significant predictor for CSS.

**Conclusions:**

Although SLR is not inferior to lobectomy in terms of oncological outcomes for patients with stage IA LCNEC, more lymph nodes can be dissected or sampled during lobectomy. Lobectomy should still be considered as a standard procedure for patients with early-stage LCNEC who are able to withstand lobectomy.

## Introduction

Pulmonary large cell neuroendocrine carcinoma (LCNEC), first reported in 1991, is a rare (around 3% of all lung cancer) but aggressive subtype of lung tumors (5-year overall survival [OS] rate is only 16.2%) (1–3). On the basis of the latest WHO classification of lung tumors, LCNEC is eliminated from the cluster of large cell carcinoma and is regrouped into the high-grade neuroendocrine tumor (NET) along with small cell lung cancer (SCLC) (3). LCNEC and SCLC share some histological features (including rosette, trabeculae, molding of nuclei and palisading, etc.), same immunohistochemical neuroendocrine markers (including a cluster of differentiation 56, neural cell adhesion molecules, etc.), and similar oncological outcomes (including metastatic behavior and poor prognosis) (2–6). However, of note, several recent studies with the aid of the next-generation sequencing and cell-free DNA detection found that LCNEC also comprised non-SCLC (NSCLC)-like subset, which may cause the individual response to different chemotherapy regimen; and the common driver mutations, including epidermal growth factor receptor (EGFR) mutation, anaplastic lymphoma kinase (ALK) rearrangements, etc., were rarely detected (4, 7). Therefore, systemic treatment for pulmonary LCNEC remains under debate in clinical practice (4, 5, 7). Based on this fact, surgical resection is still recognized as the standard scheme for patients with early-stage pulmonary LCNEC to obtain long-term survival (8, 9).

In 1995, a pivotal randomized trial administered by the Lung Cancer Study Group (LCSG) revealed a higher local-recurrence rate and inferior OS rate in patients who received sublobar resection (SLR) (including non-anatomical wedge resection [WR] and anatomical segmentectomy) rather than lobectomy for stage I NSCLC (10). Since then, lobectomy with mediastinal lymph nodes dissection as the criteria surgical procedure even in stage IA NSCLC has been upheld in the guidelines until recently (8, 9). However, several retrospective studies have disputed this dogma by demonstrating the non-inferior oncological outcomes of SLR and lobectomy for selected patients with stage IA (with or without <2 cm) NSCLC (11–14). Also, for stage IA SCLC patients in the Surveillance, Epidemiology, and End Results (SEER) database, there is no statistical difference of cancer-specific survival (CSS) or OS between the SLR group and lobectomy group (15). Potential benefits of SLR include preserving cardiopulmonary function, reducing perioperative morbidity and mortality, and providing a chance for repetitive resections (13, 16, 17). On the other hand, a debate remains about the lower rate of local recurrence after lobectomy that might bring survival benefits, particularly in patients with a good physical status. Specifically, due to the low incidence rate of pulmonary LCNEC and the majority of first diagnosed patients in the advanced stages, the investigations on the oncological clearance of SLR and the equivalency between SLR and lobectomy among pulmonary patients with LCNEC remain scarce (2, 18). Therefore, in this present study, the data retrieved from the SEER registry was used to compare the oncological outcomes following SLR and lobectomy in stage IA pulmonary LCNEC.

## Materials and Methods

### Study Cohort

**Figure 1:**
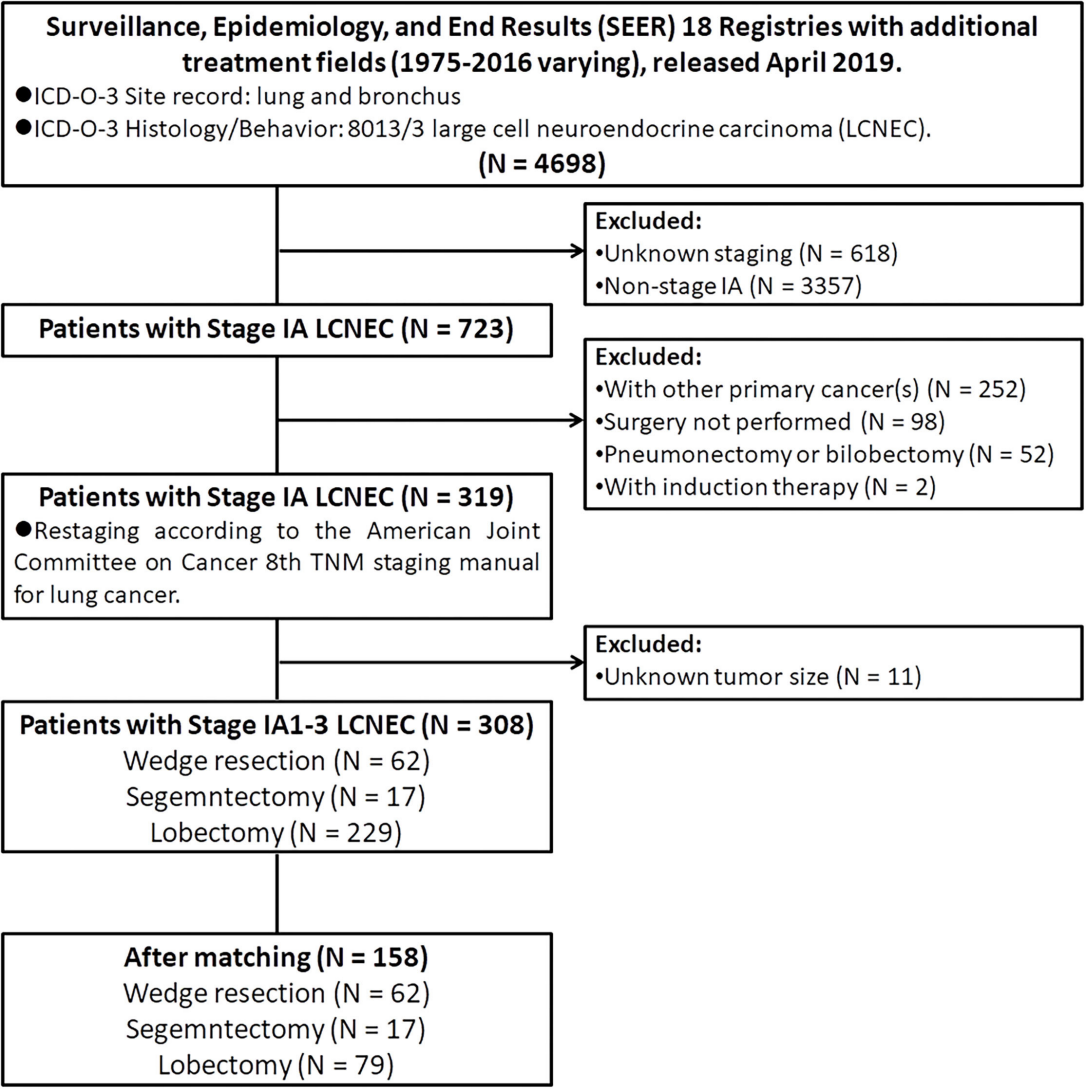
The flow diagram of the selection process for this population-based study cohort.

The public-access SEER database with additional treatment fields (1975–2016 varying) used in this study was released in April 2019, which covered ~34.6% of the US population (19). Patients diagnosed with pulmonary LCNEC (International Classification of Disease for Oncology, 3rd edition [ICD-O-3]: lung and bronchus; and ICD-O-3 histology code: 8013/3) between 1998 and 2016 were extracted by using SEER^*^Stat (version 8.3.6). Then, we limited all patients with stage IA. In addition, we also excluded patients: (I) with age at diagnosis <18 years old; (II) with other primary tumors (s); (III) did not receive surgery; (IV) received neoadjuvant or intraoperative radiation; (V) received pneumonectomy or bilobectomy; (VI) with unknown lymph nodes status; (VII) with unknown tumor size; (VIII) with incomplete follow-up data or outcomes. Finally, only 308 patients diagnosed with stage IA LCNEC who underwent WR (surgery of primary site [SPS] codes: 21), segmentectomy (SPS code: 22), or lobectomy (SPS codes: 30 and 33) were retained into the statistical analysis (Figure 1).

### Variable Definitions

The continuous variable of age at diagnosis was grouped into three groups according to the WHO recommendations on physical activity for health: <60 years old, 61–74 years old, and 75 years old and above. The year of diagnosis was divided into 4-year intervals as follows: 2001–2004, 2005–2008, 2009–2012, and 2012–2016. The insurance status of the insured, any Medicaid, and insured but no specifics, were all defined as insured; similarly, the marital status of single included never married, widowed, divorced, and single (never married). In addition, all patients were restaged to IA1, IA2, and IA3 on basis of the American Joint Committee on Cancer 8th Tumor-Nodes-Metastasis (TNM) staging manual for lung cancer.

### Outcomes

Survival time, measured in months, was calculated from the day of surgical resection to the day of death or the day of the last follow-up if the patient was documented as alive. Only patients who occurred LCNEC-related death were recorded as uncensored in CSS, but OS included any cause of death. Operative mortality was defined as any death within 30 days of surgical resection.

### Statistical Analysis

The best cutoff values for tumor size (≤20 mm/>20, ≤30 mm) and the number of lymph nodes dissection (≤5/≥6) were determined by the X-tile software (version 3.6.1, copyright Yale University 2003). The continuous variables were presented as mean ± SD and were compared by the Student's *t*-test between the SLR set and lobectomy set. For categorical variables, the chi-squared test or Fisher's exact test was applied to compare the proportions between the two sets. Survival distributions between variables were analyzed by the Kaplan–Meier method. Thereafter, potential predictors (*P* < 0.05, by the log-rank test) identified by univariate analysis for CSS and OS were enrolled into the Cox proportional hazards regression models to calculate the hazard ratios (HRs).

All of the above statistical analyses were calculated by using the SPSS software (version 24.0, IBM SPSS, Armonk, NY, USA) and the threshold value for significance was <0.05 at two sides. With aid of the R software (version 3.6.1, Mathsoft, MA, United States), propensity score matching (PSM) was implemented by the “matchit” package to reduce the potential bias between the SLR group and the lobectomy group, and the 1-to-1 nearest neighbor matching (ratio=1) was set. In addition, the survival curves before and after PSM were plotted by the “survival” and “survminer” packages.

## Results

### Patient Characteristics

A total of 308 patients diagnosed with stage IA LCNEC of the lung met inclusion criteria were finally retained in this study (Figure 1). In the demographic characteristics (Tables 1, 2), the average and the median age at diagnosis of the whole cohort were all 67 years old (range, 39–89 years old), and most of the patients were of the white race (88.6%), female (55.8%), married (54.2%), and insured (68.8%). In addition, the pulmonary LCNEC was mostly located in the right lower lobe (37.0%), followed by the right upper lobe (30.8%), left lower lobe (15.6%), right middle lobe (10.7%), and left upper lobe (5.8%).

**Table 1:** Patients' characteristics between sublobar resection and lobectomy group before propensity score matching.

**Variables**	**Sublobar resection (*n =* 79)**	**Lobectomy (*n =* 229)**	**P value**
**Age at diagnosis, (mean±SD)**	69.91 ± 9.47	65.51 ± 8.64	<0.001
**Year of diagnosis**, ***n*** **(%)**			
2001–2004	15 (19.0%)	30 (13.1%)	0.271
2005–2008	22 (27.8%)	61 (26.6%)	
2009–2012	23 (29.1%)	58 (25.3%)	
2012–2016	19 (24.1%)	80 (34.9%)	
**Race**, ***n*** **(%)**			
White	70 (88.6%)	203 (88.6%)	0.685
Black	6 (7.6%)	21 (9.2%)	
Asian or Pacific Islander	3 (3.8%)	5 (2.2%)	
**Sex**, ***n*** **(%)**			
Female	39 (49.4%)	133 (58.1%)	0.179
Male	40 (50.6%)	96 (41.9%)	
**Primary site**, ***n*** **(%)**			
Upper lobe	51 (64.6%)	158 (69.0%)	0.140
Middle lobe	2 (2.5%)	16 (7.0%)	
Lower lobe	26 (32.9%)	55 (24.0%)	
**Laterality**, ***n*** **(%)**			
Right	40 (50.6%)	140 (61.1%)	0.102
Left	39 (49.4%)	89 (38.9%)	
**Grade**, ***n*** **(%)**			
Well differentiated, I	2 (2.5%)	1 (0.4%)	0.465
Moderately differentiated, II	3 (3.8%)	6 (2.6%)	
Poorly differentiated, III	37 (46.8%)	122 (53.3%)	
Undifferentiated, IV	13 (16.5%)	33 (14.4%)	
Unknown	24 (30.4%)	67 (29.3%)	
**TNM staging**			
IA1	16 (20.3%)	36 (15.7%)	0.192
IA2	43 (54.4%)	110 (48.0%)	
IA3	20 (25.3%)	83 (36.2%)	
Tumor size (mm, mean±SD)	16.08 0.0r.21	18.19 ± 6.30	0.010
Number of lymph nodes dissection (mean±SD)	2.91 ± 4.91	8.91 ± 8.27	<0.001
**Lymph nodes dissection**, ***n*** **(%)**			
Yes	46 (58.2%)	211 (92.1%)	<0.001
No	33 (41.8%)	18 (7.9%)	
**Radiation**, ***n*** **(%)**			
Radiation after surgery	3 (3.8%)	11 (4.8%)	0.711
No radiation	76 (96.2%)	218 (95.2%)	
**Chemotherapy**, ***n*** **(%)**			
Yes	8 (10.1%)	32 (14.0%)	0.380
No/unknown	71 (89.9%)	197 (86.0%)	
**Insurance**, ***n*** **(%)**			
Any insured	53 (67.1%)	159 (69.4%)	0.416
Uninsured	0 (0.0%)	4 (1.7%)	
Unknown	26 (32.9%)	66 (28.8%)	
**Marital status**, ***n*** **(%)**			
Single	32 (40.5%)	122 (53.3%)	0.844
Married	45 (57.0%)	100 (43.7%)	
Unknown	2 (2.5%)	7 (3.1%)	

*SD, standard deviation*.

**Table 2:** Univariate and multivariate analysis of cancer-specific survival and overall survival before propensity score matching.

**Variables**	***N***	**Univariate analysis**				**Multivariate analysis**			
		**Cancer-specific survival**		**Overall survival**		**Cancer-specific survival**		**Overall survival**	
		**5-year SR (%)**	***P*-value**	**5-year SR (%)**	***P*-value**	**HR (95%CI)**	***P*-value**	**HR (95%CI)**	***P*-value**
**Age at diagnosis**									
<60 years old	79	70.3%	0.014	64.8%	<0.001	Reference		Reference	
60–74 years old	161	68.5%		54.4%		1.210 (1.013-1.556)	0.043	1.623 (1.043-2.526)	0.032
≥75 years old	68	49.8%		33.0%		2.012 (1.109-3.650)	0.021	2.588 (1.560-4.291)	<0.001
**Year of diagnosis**									
2001–2004	45	62.1%	0.287	42.2%	0.325				
2005–2008	83	63.7%		53.0%					
2009–2012	81	62.6%		51.9%					
2012–2016	99	NR		NR					
**Race**									
White	273	65.3%	0.732	53.3%	0.470				
Black	27	53.8%		41.4%					
Asian or Pacific Islander	8	85.7%		57.1%					
**Sex**									
Female	172	69.3%	0.267	56.1%	0.141				
Male	136	59.2%		47.5%					
**Primary site**									
Upper lobe	209	69.4%	0.505	54.1%	0.966				
Middle lobe	18	51.3%		51.3%					
Lower lobe	81	56.3%		47.6%					
**Laterality**									
Right	180	58.0%	0.003	47.6%	0.046	Reference		Reference	
Left	128	74.4%		58.8%		0.512 (0.330–0.795)	0.003	0.704 (0.508–0.975)	0.035
**Grade**									
Well differentiated, I	3	100.0%	0.686	0.0%	0.937				
Moderately differentiated, II	9	60.0%		53.3%					
Poorly differentiated, III	159	64.3%		53.3%					
Undifferentiated, IV	46	63.0%		46.4%					
Unknown	91	67.1%		52.2%					
**TNM staging**									
IA1	52	61.4%	0.509	52.3%	0.160				
IA2	153	66.6%		54.9%					
IA3	103	63.7%		48.2%					
**Tumor size**									
≤ 20mm	205	65.7%	0.552	54.5%	0.102				
>20, ≤ 30mm	103	63.7%		48.2%					
**Lymph nodes dissection**									
Yes	257	66.5%	0.093	52.9%	0.120				
No	51	57.3%		49.0%					
**No. of lymph nodes dissection**									
≤ 5	156	58.5%	0.003	46.6%	0.013	Reference		Reference	
≥6	152	72.8%		59.5%		0.622 (0.385–0.994)	0.042	0.774 (0.539–1.112)	0.167
**Radiation**									
Radiation after surgery	14	58.0%	0.057	52.2%	0.506	Reference			
No radiation	294	65.3%		52.3%		0.585 (0.289–1.183)	0.135		
**Chemotherapy**									
Yes	40	78.3%	0.376	72.7%	0.078				
No/unknown	268	63.0%		49.6%					
**Insurance**									
Any insured	212	65.4%	0.506	54.2%	0.479				
Uninsured	4	50.0%		50.0%					
Unknown	92	64.3%		47.5%					
**Marital status**									
Single	132	70.2%	0.123	55.5%	0.487				
Married	167	59.9%		49.2%					
Unknown	9	100.0%		80.0%					
**Resection**									
Sublobar resection	79	56.5%	0.037	42.9%	0.019	Reference		Reference	
Lobectomy	229	67.8%		55.7%		0.585 (0.289–1.183)	0.135	0.815 (0.560–1.186)	0.285

*SR, survival rate; No., number; HR, hazard ratio; CI, confidence interval; NR, no reached*.

In the surgical operation (Tables 1, 2), 229 patients underwent lobectomy, 62 patients underwent WR, and only 17 patients underwent segementectomy. In total, 83.4% of the patients (257/308) received at least one lymph node dissection (mean, 7.37; range, 0–55). In the pathological examination, the mean tumor size was 17.65 millimeters (mm; range, 7–30 mm), and the patients diagnosed with stage T1a, T1b, and T1c were 52 cases, 153 cases, and 103 cases, respectively. Poorly differentiated and undifferentiated LCNEC observed under a microscope accounted for the majority (51.6 and 14.9%, respectively). In the systemic treatment, 14 patients (including 3 WRs and 11 lobectomies) received postoperative radiation and 40 patients (including 8 WRs and 32 lobectomies) received chemotherapy.

### SLR vs. Lobectomy

Patients who underwent lobectomy were younger (65.51 5.5.64 vs. 69.91 9.5.47, *P* < 0.001), but showed larger tumor size (18.19 8.1.30 vs. 16.08 0.1.21, *P* = 0.010) than those who underwent SLR. In addition, the percentage of patients receiving lymph nodes dissection in the SLR group was significantly lower than that in the lobectomy group (58.2 vs. 92.1%, *P* < 0.01); similarly, patients with SLR had a less number of lymph nodes dissected (2.91 0.9e.91 vs. 8.91 89e27, *P* < 0.001). The differences between the two groups are further detailed in Table 1. In addition, three patients occurred death within 30 days after lobectomy and only one patient occurred operative mortality after SLR (*P* = 0.976).

**Figure 2:**
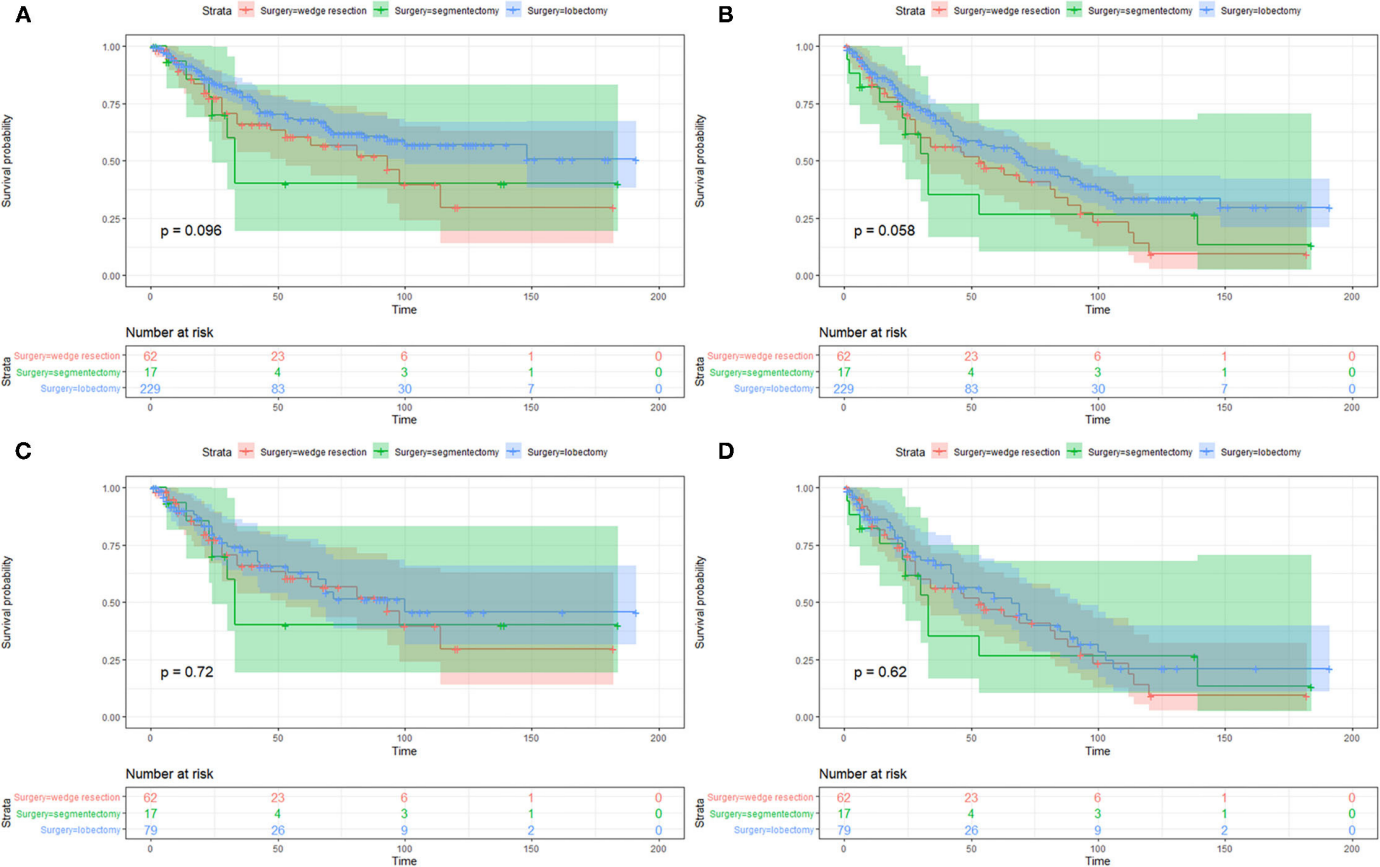
Kaplan–Meier survival curves of cancer-specific survival **(A,C)** and overall survival **(B,D)** between patients with wedge resection, segmentectomy, and lobectomy before and after propensity score matching.

On the Kaplan–Meier survival analysis, patients who underwent SLR showed worse 5-year CSS rate and OS rate (56.5 vs. 67.8%, *P* = 0.037; 42.9 vs. 55.7%, *P* = 0.019; Table 2). Separately, the CSS in patients with WR or segmentectomy was worse than those with lobectomy in trend (WR: 60.4 vs. 67.8%, *P* = 0.084; segmentectomy: 40.0 vs. 67.8%, *P* = 0.095; Figure 2A). However, WR and segmentectomy were significantly associated with worse 5-year OS rate compared to lobectomy (WR: 46.9 vs. 55.7%, *P* = 0.023; segmentectomy: 26.5 vs. 55.7%, *P* = 0.083; Figure 2B). Unexpectedly, after the Cox multivariate regression analysis, there was no significant difference between the SLR group and the lobectomy group in CSS or OS (all *P* > 0.05, Table 2).

The imbalanced variables, including age at diagnosis, tumor size, and lymph nodes dissection (yes/no), were included in the 1-to-1 PSM. After that, a total of 158 patients were selected, with 79 patients in both the SLR group and lobectomy group. No significant difference was observed between the two groups in 13 of the 15 variables after PSM, except for whether to dissect lymph nodes and the number of lymph nodes dissection (Supplementary Table S1). On the Kaplan–Meier survival analysis, there was no significant difference of CSS or OS between the two matched groups as well (all *P* > 0.05, Figures 2C,D).

### Prognostic Factors for CSS and OS

**Figure 3:**
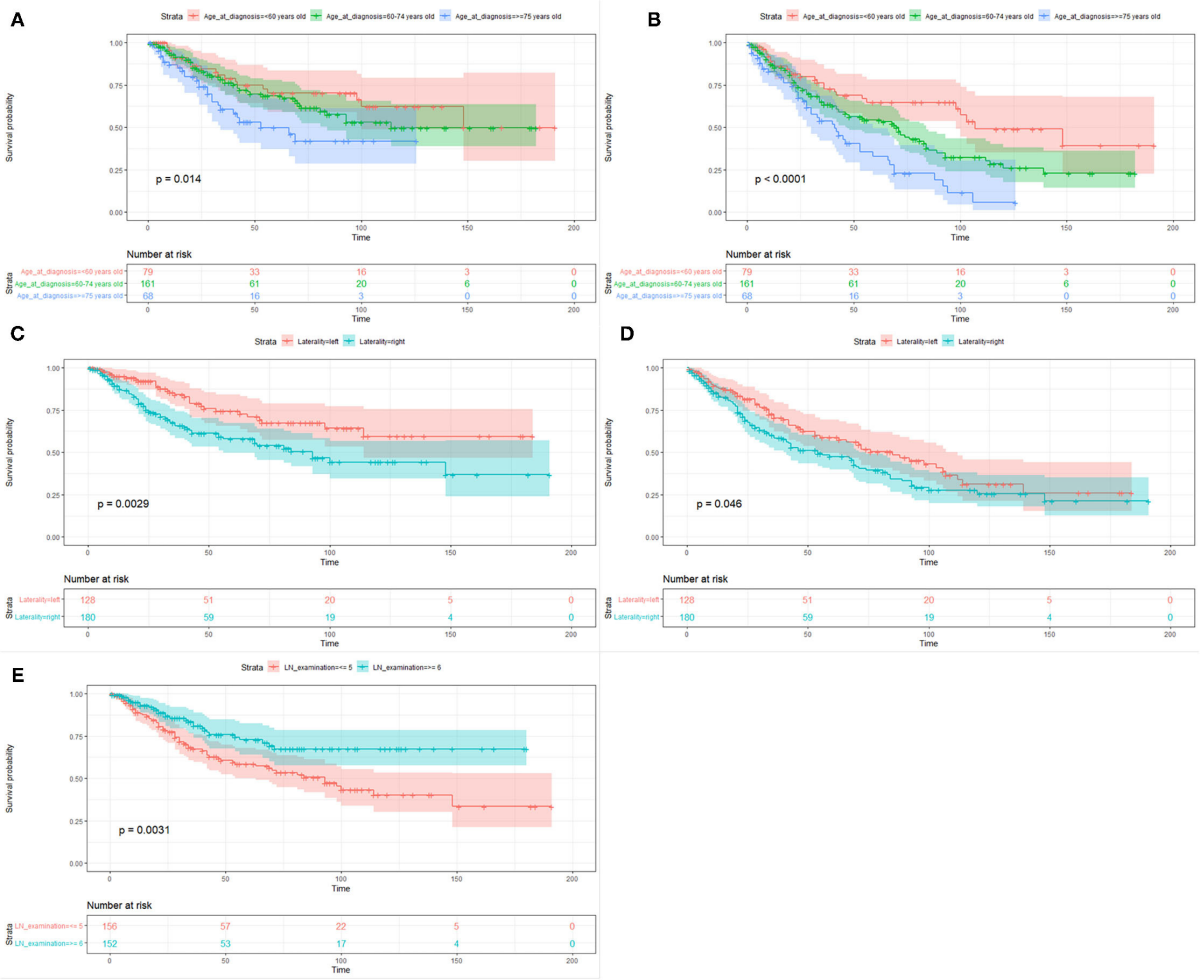
Kaplan–Meier survival curves of cancer-specific survival **(A,C,E)** and overall survival **(B,D)** based on age at diagnosis, laterality of tumor, and the number of lymph nodes dissection.

The median follow-up time of the 308 patients was 33.5 months (range from 1 to 191 months). The 3-year and 5-year CSS and OS rates were 73.5 and 63.6% and 64.9 and 52.4%, respectively. Table 2 showed the univariate and multivariate analyses. The age at diagnosis (Figures 3A,B) and laterality of tumor (Figures 3C,D) were identified as independent prognostic factors for CSS and OS. In addition, the number of lymph nodes dissection <5 was a risk factor for CSS (Figure 3E), but not for OS.

## Discussion

The present study was conducted to investigate the CSS and OS associated with SLR and lobectomy in 308 patients with stage IA LCNEC retrieved from the National Cancer Institute's SEER database, by using the Cox proportional hazards regression model and PSM to adjust the potential confounding factors. We found that SLR was not inferior to lobectomy for CSS and OS. However, multivariate analyses demonstrated that advancing age and right-sided lung resection were significant risk factors for CSS and OS; in addition, patients receiving lymph nodes dissection <5 were associated with decreased CSS.

In line with the published literature on patients with stage IA LECNEC of the lung who underwent lobectomy (27.0–67.0%), the 5-year OS rate was 67.8% in this population-based study, which was worse than other NSCLC (18, 20–23). However, owing to the low incidence and a lack of evidence-based surgical treatment for LCNEC, there was a limited study to evaluate the survival after SLR (6, 18, 22, 24). As reported by the Neuroendocrine Tumors (NETs) Working Group, 24 patients collected from eight high-volume European Thoracic Surgery Institutions received SLR, including 13 WR and 11 lobectomies; furthermore, compared to 81 patients who received lobectomy, these patients received SLR showed an equivalent 5-year OS rate (6). A similar survival outcome was verified by another single-institution study, which was the largest study cohort between 1991 and 2006 (22). Conversely, in 2019, a prognostic analysis comparing SLR (133 cases) vs. lobectomy or bilobectomy (425 cases) utilizing the SEER database found the 5-year OS rate was 22.5 and 42.5%, respectively (*P* < 0.001) (24). However, of note, patients diagnosed with stage I through IV were all enrolled in the above studies and most of the statistical analyses were performed on small size samples.

Recently, Waseem et al. identified 1,011 patients diagnosed with stage I LCNEC (≤3 cm) from the National Cancer Database (NCDB) between 2004 and 2014, and the findings showed patients who underwent lobectomy had better OS when compared with those with SLR (before PSM: 56.6% vs. 37.9%, *P* < 0.001; after PSM: 60.3% vs. 41.5%, *P* = 0.001), which was the largest study cohort for stage I LCNEC until now (18). The constituent ratio of WR, segmentectomy, and lobectomy for stage IA LCNEC was parallel between the NCDB database and the SEER database (22.1, 4.0, and 74.0% vs. 20.1, 5.5, and 74.4%, respectively). In addition, fully consistent with our findings, patients receiving SLR were more likely to have increased age, smaller size, and less likely to have lymph nodes dissection than those receiving lobectomy. However, SLR showed no inferiority to lobectomy for CSS and OS of stage IA LCNEC by the multivariate analysis and propensity score analysis in this study. We speculate that only the PSM performed in the study of Waseem et al. may strengthen the power to identify the statistical difference in survival. As known, if the ratio of the patients in the control group (lobectomy) to the patients in the study group (SLR) is <10-to-1, plenty of patients in the study group could not match the nearest control cases during the process of PSM, which may increase the man-made selection bias (25). Therefore, it is necessary to conduct the multivariate analysis to adjust potential confounding factors before PSM and/or after PSM (2, 13, 23, 25). Additionally, the reasons for SLR include intention-to-treat and compromise-to-treat in clinical practice, and the intentional SLR is appropriate for patients with poor pulmonary reserve or other major comorbidities; moreover, the sufficient parenchymal resection margin is vital when surgeons perform the SLR (26). Regrettably, these variables were not available within the SEER database. On the whole, the above weaknesses in the published and our studies may preliminarily explain why the results were different between the two large size samples, and the debate on the oncological outcomes following SLR compared to lobectomy for patients with stage IA LCNEC remains (6, 18, 22, 24).

We observed a notable difference in the number of lymph nodes dissected by lobectomy and SLR, and the proportion of the patients without lymph nodes dissection in the SLR group was significantly higher than that in the lobectomy group, which were fully consistent with other studies for comparing lobectomy and SLR for stage IA NSCLC based on the NCDB (14, 27). It meant that patients receiving SLR for stage IA lung cancer in the NCDB and SEER database may not receive adequate lymph nodes dissection. Previous studies have found that adequate lymph nodes examination for lung cancer, especially in SLR, was associated with more accurate pathological staging and better survival (14, 27, 28). Similarly, our result showed that the number of lymph nodes dissection >6 yielded improved long-term benefit in CSS for stage IA LCNEC patients, and more lymph nodes dissection did not increase the operative mortality. In addition, in line with other studies on early-stage LCNEC, Cox regression analysis revealed advancing age to be associated with worse survival (23).

Right-side radiation or pneumonectomy for lung cancer was regarded as an independent risk factor for long-term survival (29, 30). The study on the right ventricular (RV) response to lung resection by using cardiovascular MRI, reported by Philip et al., found that RV dysfunction occurred immediately following lung resection, especially right-sided resection, and persisted two months or more, which may be associated with the dyspnea and reduced functional capacity (29). In addition, Carolyn et al. reported that RV end-diastolic volume and center venous pressure would significantly be increased after right-sided lung resection as well (30). Therefore, the right-sided lung resection for early-stage LCNEC may also have a negative impact on the cardiopulmonary function, postoperative complications, and long-term survival, which was similar to a prognostic analysis in patients with stage IA SCLC (15).

Undeniably, this population-based study had several limitations. First, the prospective study was difficult to carry out due to the rarity of early-stage LCNEC, therefore, this study was conducted from a retrospective viewpoint. However, multivariate analysis and PSM were performed to reduce the confounding factors and selection bias. Second, several important variables associated with the oncological outcomes after limited resection, such as the reason for SLR, preoperative cardiopulmonary function, the status of resection margin, local recurrence, etc., were not documented in the SEER database. Third, limited by the number of patients who underwent SLR for stage IA LCNEC, we could not further compare the outcomes of segmentectomy and WR.

Although SLR is not inferior to lobectomy in terms of survival for patients with stage IA LCNEC of the lung, more lymph nodes can be dissected for more accurate staging during lobectomy. Moreover, it is difficult to make a definitive diagnosis of LCNEC during the intraoperative cytology or frozen section. However, the histological features of NETS under the microscope are easily observed, and lobectomy should be performed for these patients according to the advanced guidelines. On a whole, lobectomy should still be considered as a standard procedure for patients with early-stage LCNEC of the lung who are able to withstand lobectomy.

## Data Availability Statement

The raw data supporting the conclusions of this article will be made available by the authors, without undue reservation.

## Ethics Statement

The studies involving human participants were reviewed and approved by the Surveillance Research Program in National Cancer Institute, Division of Cancer Control and Population Science (DCCPS) (Reference No. 12101-Nov2018) and the board-certified Research Ethics Committee at Sun Yat-sen University Cancer Center (No. B2018-011). Written informed consent for participation was not required for this study in accordance with the national legislation and the institutional requirements.

## Author Contributions

XY, MZ, and FW: conceptualization, writing-original draft, and software and conceptualization. XY and HZ: data curation, methodology, and preparation. HX, CH, LD, and RJ: visualization and investigation. XG, KM, LW, LZ, and ZY: writing–reviewing and editing and supervision. XY, MZ, FW, and YY: software and validation. XY, HZ, HX, and RJ: formal analysis. LZ and ZY: supervision and conceptualization. All authors contributed to the article and approved the submitted version.

## Funding

This study was funded by the Shenzhen Key Medical Discipline Construction Fund (No. SZXK075) and the Sanming Project of Medicine in Shenzhen (No. SZSM201612097).

## Conflict of Interest

The authors declare that the research was conducted in the absence of any commercial or financial relationships that could be construed as a potential conflict of interest.

## Publisher's Note

All claims expressed in this article are solely those of the authors and do not necessarily represent those of their affiliated organizations, or those of the publisher, the editors and the reviewers. Any product that may be evaluated in this article, or claim that may be made by its manufacturer, is not guaranteed or endorsed by the publisher.

## Abbreviations

LCNEC: large cell neuroendocrine carcinoma
OS: overall survival
CSS: cancer-specific survival
SLR: sublobar resection
WR: wedge resection
WHO: World Health Organization
NET: neuroendocrine tumor
SCLC: small cell lung cancer
NSCLC: non-small cell lung cancer
DNA: deoxyribonucleic acid
EGFR: epidermal growth factor receptor
ALK: anaplastic lymphoma kinase
SEER: the Surveillance, Epidemiology, and End Results
ICD-O-3: International Classification of Disease for Oncology, 3rd edition
PSM: propensity score matching.
