# Supplementary material for: Survival After Lobectomy vs. Sublobar Resection for Stage IA Large-Cell Neuroendocrine Carcinoma of the Lung: A Population-Based Study
Source: Front Surg. 2022 Mar 15;9:856048. doi: 10.3389/fsurg.2022.856048 (PMC8964597; doi:10.3389/fsurg.2022.856048)
Supplement: Supplementary file 1 [file Table_1.DOCX]

**Table S1** Patients’ characteristic between sublobar resection group and lobectomy group after propensity score analysis.

| **Variables** | **Sublobar resection (n=79)** | **Lobectomy (n=79)** | **P value** |
| --- | --- | --- | --- |
| **Age at diagnosis, (mean±SD)** | 69.91±9.47 | 69.20±8.68 | 0.624 |
| **Year of diagnosis, n (%)** |  |  |  |
| 2001-2004 | 15 (19.0%) | 10 (12.7%) | 0.119 |
| 2005-2008 | 22 (27.8%) | 25 (31.6%) |  |
| 2009-2012 | 23 (29.1%) | 14 (17.7%) |  |
| 2012-2016 | 19 (24.1%) | 30 (38.0%) |  |
| **Race, n (%)** |  |  |  |
| White | 70 (88.6%) | 69 (87.3%) | 0.448 |
| Black | 6 (7.6%) | 9 (11.4%) |  |
| Asian or Pacific Islander | 3 (3.8%) | 1 (1.3%) |  |
| **Sex, n (%)** |  |  |  |
| Female | 39 (49.4%) | 51 (64.6%) | 0.054 |
| Male | 40 (50.6%) | 28 (35.4%) |  |
| **Primary site, n (%)** |  |  |  |
| Upper lobe | 51 (64.6%) | 55 (69.6%) | 0.165 |
| Middle lobe | 2 (2.5%) | 6 (7.6%) |  |
| Lower lobe | 26(32.9%) | 18 (22.8%) |  |
| **Laterality, n (%)** |  |  |  |
| Right | 40 (50.6%) | 49 (62.0%) | 0.149 |
| Left | 39 (49.4%) | 30 (38.0%) |  |
| **Grade, n (%)** |  |  |  |
| Well differentiated, I | 2 (2.5%) | 0 (0.0%) | 0.362 |
| Moderately differentiated, II | 3 (3.8%) | 1 (1.3%) |  |
| Poorly differentiated, III | 37 (46.8%) | 46 (58.2%) |  |
| Undifferentiated, IV | 13 (16.5%) | 11 (13.9%) |  |
| Unknown | 24 (30.4%) | 21 (26.6%) |  |
| **TNM staging** |  |  |  |
| IA1 | 16 (20.3%) | 36 (15.7%) |  |
| IA2 | 43 (54.4%) | 110 (48.0%) |  |
| IA3 | 20 (25.3%) | 83 (36.2%) |  |
| **Tumor size (mm, mean±SD)** | 16.08±6.21 | 15.63±5.77 | 0.643 |
| **Number of lymph nodes dissection (mean±SD)** | 2.91±4.91 | 8.23±9.59 | <0.001 |
| **Lymph nodes dissection, n (%)** |  |  |  |
| Yes | 46 (58.2%) | 61 (77.2%) | 0.011 |
| No | 33 (41.8%) | 18 (22.8%) |  |
| **Radiation, n (%)** |  |  |  |
| Radiation after surgery | 3 (3.8%) | 2 (2.5%) | 0.649 |
| No radiation | 76 (96.2%) | 77 (97.5%) |  |
| **Chemotherapy, n (%)** |  |  |  |
| Yes | 8 (10.1%) | 9 (11.4%) | 0.797 |
| No/unknown | 71 (89.9%) | 70 (88.6%) |  |
| **Insurance, n (%)** |  |  |  |
| Any insured | 53 (67.1%) | 52 (65.8%) | 0.866 |
| Uninsured | 0 (0.0%) | 0 (0.0%) |  |
| Unknown | 26 (32.9%) | 27 (34.2%) |  |
| **Marital status, n (%)** |  |  |  |
| Single | 32 (40.5%) | 38 (48.1%) | 0.521 |
| Married | 45 (57.0%) | 38 (48.1%) |  |
| Unknown | 2 (2.5%) | 3 (3.8%) |  |

SD, standard deviation.
